# [Expression of Concern] c-Fos/ERK promotes the progression from pancreatic intraepithelial neoplasia to pancreatic ductal adenocarcinoma 

**DOI:** 10.3892/or.2026.9092

**Published:** 2026-03-05

**Authors:** Lei You, Xiaoxia Ren, Yongxing Du, Wenjing Zhao, Ming Cui, Ge Chen, Yupei Zhao

Oncol Rep 36: 3413–3420, 2016; DOI: 10.3892/or.2016.5169

Following the publication of the above paper, it was drawn to the Editor's attention by a concerned reader that, regarding the histological images shown in Fig. 2B on p. 3416, the ‘Caerulein 7 days’ and ‘Caerulein 14 days’ panels showed an overlapping section, with the image also being magnified in the latter panel relative to the first. In addition, the immunohistochemical images shown for the ‘c-Fos/PK, caerulein+U0126’ experiment in Fig. 3A and the ‘c-Fos/Control/Caerulein 7 days’ experiment in Fig. 4A were identical, suggesting that at least one of these figures had been assembled incorrectly.

The authors have been contacted by the Editorial Office to offer an explanation for these apparent anomalies in the presentation of the data in this paper. Owing to the fact that the Editorial Office has been made aware of potential issues surrounding the scientific integrity of this paper, we are issuing an Expression of Concern to notify readers of these potential problems while the Editorial Office continues to investigate this matter further.

